# Solvatochromism, Acidochromism and Photochromism of the 2,6-Bis(4-hydroxybenzylidene) Cyclohexanone Derivative

**DOI:** 10.3390/ijms24065286

**Published:** 2023-03-09

**Authors:** Mihaela Homocianu, Diana Serbezeanu, Vlad Bubulac Tachita

**Affiliations:** “Petru Poni” Institute of Macromolecular Chemistry, 41A, Grigore Ghica Voda Alley, 700487 Iasi, Romania

**Keywords:** 2,6-bis(4-hydroxybenzylidene) cyclohexanone, intramolecular charge transfer, solvatochromism, acidochromism, photochromism

## Abstract

In this paper, we studied the photophysical behavior of 2,6-bis(4-hydroxybenzylidene) cyclohexanone (BZCH) under the influence of various stimuli. The photophysical properties were correlated with different solvent parameters, such as the Kamlet–Abraham–Taft (KAT), Catalán, and Laurence solvent scales, suggesting that the behavior of BZCH is influenced by both nonspecific and specific solvent-solute interactions. The Catalán solvent dipolarity/polarizability parameters were found to have a significant role in the solvatochromic behavior, which is also confirmed by the KAT and Laurence models. The acidochromism and photochromism properties of this sample in dimethylsulfoxide and chloroform solutions were also investigated. The compound showed reversible acidochromism after the addition of dilute NaOH/HCl solutions, accompanied by a change in color and the appearance of a new absorption band (514 nm). The photochemical behavior was also examined by irradiating BZCH solutions with both 254 and 365 nm light.

## 1. Introduction

Photochromic materials have gained great interest recently due to their unique photoresponsive behavior and promising applications as smart materials [1,2].

Chalcone derivatives containing the ketoethylenic group (-CO-CH=CH-) provide a rich source for the synthesis of optically active compounds [3]. These derivatives have great potential for applications due to their specific electronic structures (i.e., a completely delocalized electron system) [4], photophysical properties [5,6,7], and biological activities, such as being antimicrobial [8], antioxidant [9], anticonvulsant [10], inhibitors of inflammatory markers [11], and antifungal agent [12]. In recent years, dibenzylidene cyclohexanone derivatives have been used as corrosion inhibitors [13], fluorescent sensors for detecting Hg^2+^ [14], and chromium ions in aqueous media [15]. Alejo-Armijo et al. [16] recently investigated the multistate species of 2,6-bis(arylidene) cyclohexanones and their behavior at different pH values using NMR, UV-vis, and flash photolysis methods. They found that the colorless compound, 7,8-dihydro-6H-chromeno [3,2-d] xanthene was isolated from acidic solutions up to a neutral range. Moreover, when the pH environment changed, the 2,6-bis(4-hydroxybenzylidene) cyclohexanone (curcumin-resembling molecular probe) [17] showed appropriate properties for colorimetric and fluorometric assay. A significant acidochromic behavior was observed for 2,6-bis(4-dimethylamino-benzylidene)-cyclohexanone [18] after titration with hydrochloric acid (HCl) in ethanol solution. This behavior is due to the preferential protonation of the chromophoric N,N-dimethylamino group, which is due to the formation of a quaternary salt and deactivation of the resonance system, which was confirmed by computational studies.

The study of photochromic compounds in different solvents can provide advanced knowledge about their photophysical behavior due to specific solute-solvent interactions and may open new doors to the formation of new species or the possibility of the stabilization of species from a network of chemical reactions that are difficult or even impossible to observe in other studies. The influence of solvent multiparameters on spectral properties has been extensively studied and correlated to the solute behavior in solution [19]. The parameters introduced by Reichardt ($E_{T}^{N}$) [20], KAT, and Catalán for all solvents have been widely used as indicators for analyzing spectral changes due to the solvent and those resulting from both specific or non-specific solute-solvent interactions [21].

Previous studies [18,22] have shown that various 2,6-dibenzylidene-cyclohexanone derivatives (substituted with H, p-Cl, p-NO_2_, p-OCH_3_, p-N(CH_3_)_2_) exhibit acidochromic behavior and biological activity [22]. It was observed that protonation/deprotonation of the cyclohexanone derivatives substituted with phenylphosphonic dichloride, phenyl dichlorophosphate, terephthaloyl chloride, or terephthaloyl-bis-(4-oxybenzoylchloride) leads to the formation of a new absorption band and a change in the color of the solution [23]. The detailed electronic properties and their relationship with environmental parameters (acidity, basicity, polarity, etc.) provide important information necessary for understanding the biological activity of these compounds.

**Figure 1:**
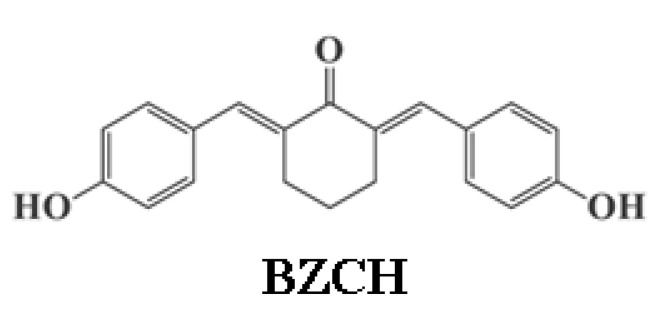
Chemical structure of 2,6-bis(4-hydroxybenzylidene) cyclohexanone (BZCH).

In this study, the influence of the microenvironment on the photophysical properties of 2,6-bis(4-hydroxybenzylidene) cyclohexanone (BZCH), (Figure 1) was investigated using a multiparametric linear regression analysis based on the Kamlet–Abraham–Taft (KAT), Catalán, and Laurence empirical solvent scales. Our research covers a wide group of solvents (15) including nonpolar and polar solvents. Moreover, knowledge of the solvatochromic, acidochromic, and photochromic behavior of BZCH derivatives is fundamental for evaluating their prospective use in future applications. The relative contribution of specific and non-specific interactions to the photophysical properties was investigated using the KAT, Catalán, and Laurence solvation energy relationships. Subsequently, the effects of adding dilute NaOH/HCl solutions and exposure to 254 and 365 nm UV light on the absorption and emission behavior were then analyzed and described in detail.

## 2. Results and Discussion

### 2.1. Solvatochromic Analysis

**Figure 2:**
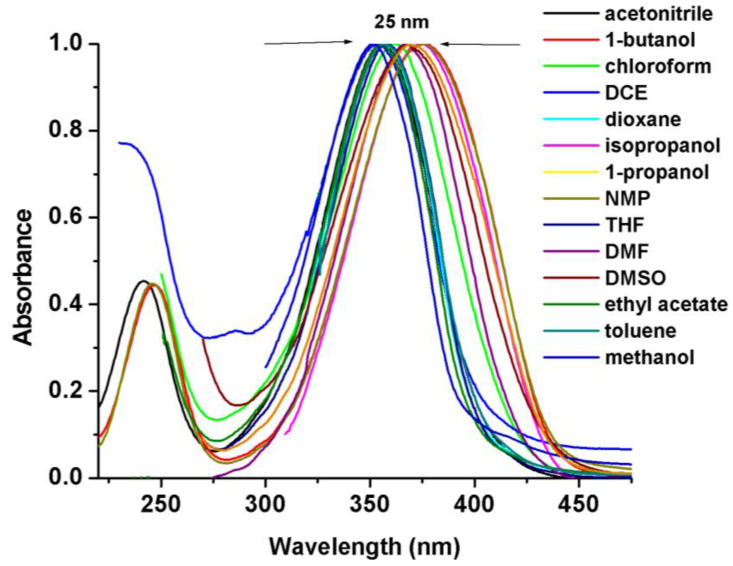
Normalized UV-Vis absorption spectra of BZCH in selected solvents.

We investigated changes in the absorption spectra of BZCH in a series of fifteen solvents with varying polarities, ranging from nonpolar to polar solvents (Table 1). Figure 2 shows the selective absorption spectra for this compound, with the values of the absorption spectral maxima listed in Table 1. As the polarity of the solvents increases, changes can be seen in the shape, intensity, and wavelengths of absorption maxima, which are a result of intramolecular interactions between the donor and acceptor groups in the ground state. In all solvents used, BZCH exhibited only a single absorption band with a maximum wavelength (λ_max_) ranging from 351 to 376 nm, except in solutions with ACN, 1-butanol, and 1-propanol, where a weak absorption band around 240 nm was observed due to their high transparency. The main absorption band can be attributed to π-π* and n-π* transitions, which are caused by the presence of double bonds and a lone pair of electrons on the oxygen atoms. A redshift (bathochromic effect) is observed in the absorption spectra when going from 1-propanol (374 nm) to toluene (351 nm, Δλ = λ_1-propanol_ − λ_toluene_ = 25 nm, as shown in Table 1 and Figure 2. These bathochromic shifts occur when the solvent stabilizes the excited state more than the ground state.

Numerous multiparametric equations have been developed to investigate the effects of solvents on spectral data. To study the changes in the absorption spectra recorded in solvents with different polarities, we analyzed the spectral data using the following multiparametric solvent scales proposed by KAT [24], Catalán [25], and Laurence [26], whose parameters are listed in Table 2. Thus, the values of the absorption maxima (${\tilde{\nu }}_{\exp}^{\max}$) and transition energies (E_T_) of BZCH were correlated with the descriptors of all solvents, given in the KAT, Catalán, and Laurence (Equations (1)–(3)) empirical scales, using the following equations:

(1) $${\tilde{\nu }}_{{}_{\exp}}^{\max}={\tilde{\nu }}_{\max,}{}_{0}+a_{1}\alpha +b_{1}\beta +c_{1}\pi$$

where ${\tilde{\nu }}_{\exp}^{\max}\;$ is the solvent-dependent property, π* is a measure of the dipolarity/polarizability of the solvents, *α* is the acidity of the hydrogen bond donor (HBD), and *β* is the basicity of the hydrogen bond acceptor (HBA).

(2) $${\tilde{\nu }}_{\exp}^{\max}={\tilde{\nu }}_{\max,}{}_{0}+a_{2}SA+b_{2}SB+c_{2}SP+d_{2}SdP$$

where *SA* is acidity, *SB* is basicity, *SP* is polarizability, and *SdP* is solvent dipolarity;

(3) $${\tilde{\nu }}_{{}_{\exp}}^{\max}={\tilde{\nu }}_{\max,}{}_{0}+a_{3}DI+b_{3}ES+c_{3}\alpha_{1}+d_{3}\beta_{1}$$

where *DI* refers to the dispersion and induction interactions, and *ES* refers to the electrostatic interactions between the permanent dipoles of the solute and solvent. The variables *α*_1_ and *β*_1_ represent the hydrogen bond donating (HBD) and accepting (HBA) abilities of the solvents, respectively.

The regression coefficients {a_1_-a_3_, b_1_-b_3_, c_1_-c_3_, and d_2_ and d_3_} reflect the influence of each solvent parameter on the spectral properties of the BZCH compound, as obtained through multiple regression analysis. The size and sign of the coefficients indicate the magnitude and direction of the effect of the corresponding solvent-solute interactions on the electronic transition energies. The experimental absorption spectral data obtained in 15 solvents with various polarities were used in this analysis. The results of the multiple linear analysis are summarized in Table 3. 

A negative sign of the obtained regression coefficients (Table 3) indicates bathochromic shifts with increasing solvent polarizability and suggests that the electron excited state is being stabilized compared to the ground state. A weak correlation (R^2^ = 0.775) between the absorption maxima (${\tilde{\nu }}_{\exp}^{\max}$) and KAT solvent parameters is due to non-specific solvent effects making a significant contribution to the spectral shifts. The dominant solvent property affecting the absorption band (${\tilde{\nu }}_{\exp}^{\max}$) of BZCH is the solvent polarity/polarizability (π*—46% relative contribution), with HBD (*α*—30% relative contribution) playing a much smaller role, followed by HBA (*β*—24% relative contribution), as shown in Table 4. Therefore, this confirms that the polarity of the solvent is a major influencing factor, as seen in the low values for R^2^ (0.441 and 0.221) obtained in the multiple regression fits without considering π* (Equations (S1) and (S2), in the Supplementary Materials).

In the case of the Catalán model, the polarizability of the solvent (Table 4) is the most important factor affecting the spectral shifts, and the contribution of dipolarity (*SdP*) is almost insignificant, as shown by the large deviation of the coefficient (d_2_, Table 3). Eliminating the contributions of the *SdP* parameters has no significant effect on the resulting correlations, with a new R^2^ = 0.940. Both methods accurately predict the predominant effect of the dipolarity/polarizability of the solvent on the solvatochromism of the studied compound. In fact, the removal of the *SP* and *SdP* contributions in Equation (S9) (Supplementary Materials) leads to a minimal decrease in the correlation coefficient from R^2^ = 0.940 (Equation (S10)) to 0.859 (Equation (S9)).

Based on the Laurence solvent scale, determined both experimentally and theoretically, we found that non-specific parameters (*DI* + *ES* = 70.2%) and solvent basicity (*β*_1_) also have a significant effect on the solvatochromism of BZCH absorption, while solvent acidity (*α*_1_) has a negligible effect (Table 4). The Catalán scale provides better correlations than the KAT and Laurence models, as evidenced by the higher correlation coefficient values (R^2^ = 0.961, Table 3). Moreover, the sensitivity of BZCH molecules to solvent polarity was confirmed by all solvent scales used (with Laurence having the highest effect (62.45% for *DI*) followed by KAT (45.57% for π*) and Catalán (41.77% for *SP*). This indicates that solvent polarizability/dipolarity plays a more important role than solvent acidity or basicity. In order to visualize the quality of the fits, we present graphs (Figure S1) that show the correlation between the calculated absorption maxima (${\tilde{\nu }}_{\exp}^{\max}$, calculated according to Equations (1)–(3) and the regression coefficients) versus the corresponding experimental values (${\tilde{\nu }}_{\exp}^{\max}$, Table 1). This resulted in a linear correlation (Figure S1a–c) with positive values for the slope between the calculated and experimentally determined absorption maxima. Thus, it was found that the data resulting from the linear multiple regression is in agreement with the experimental data.

### 2.2. Basichromism of 2,6-Bis(4-hydroxybenzylidene) Cyclohexanone

**Figure 3:**
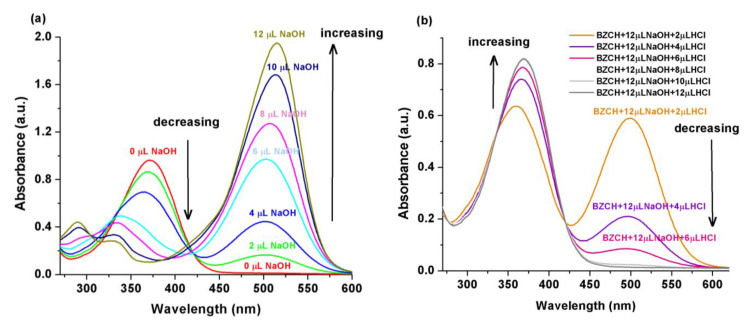
Absorption spectral changes of BZCH in DMSO induced by the titrations with dilute (0.1 N) NaOH (**a**) and followed by the addition of dilute HCl solutions (**b**).

The behavior of the BZCH sample in the presence of the various incremental amounts of the dilute NaOH/HCl (0.1 N) solutions was evaluated by monitoring changes in its UV-Vis absorption and fluorescence spectra. Figure 3a shows the transformation of the BZCH sample absorption spectra in DMSO solution upon the successive addition of dilute NaOH. As the amount of NaOH increases, the absorption maximum at 369 nm gradually decreases and a new band gradually appears at 514 nm (corresponding to the deprotonated species), which rises in intensity. The position of the main absorption band (369 nm) was also affected by changes in the solution pH, i.e., the λ_max_ is hypsochromically shifted by approximately 80 nm (to a shorter wavelength) and a new shoulder appears at 333 nm after the additions of 12 µL NaOH (Figure 3a). Moreover, the absorbance at 369 nm is decreased after the addition of 12 µL NaOH (increasing the pH), which indicates that the reactive ketoethylene group (-CO-CH=CH-) is in its unprotonated form [23]. Then, the resulting solution was protonated by gradually titrating it with dilute HCl, when, assuming that due to the speed of the processes occurring in this solution, a sudden decrease in absorbance was observed upon the addition of 2, 4, and 6 µL of HCl (Figure 3b). As noted in our previous work [23], a similar acidochromic behavior was observed for the two polyesters containing 2,6-bis(2-hydroxyarylidene) cyclohexanone in the presence of NaOH solutions, but with only modest changes in absorbance intensity after the addition of 12 µL NaOH compared to the behavior of the BZCH sample. Badal et al. [18] also reported significant acidochromic behavior for 2,6-bis(4-dimethylamino-benzylidene) cyclohexanone.

**Figure 4:**
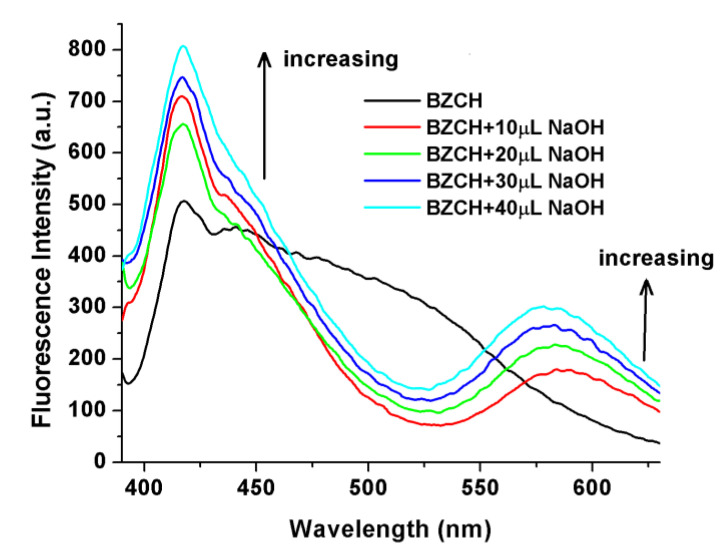
Fluorescence spectra of pure BZCH in DMSO solution and after the addition of dilute NaOH solutions. Excitation wavelength used was the corresponding absorption maximum.

Furthermore, the fluorescence spectral changes that result from an incremental increase in the concentration of NaOH in the BZCH in DMSO solvent were studied, as depicted in Figure 4. With increasing NaOH concentration, there is a remarkable enhancement of fluorescence at λ_em_ = 418 nm, accompanied by the development of a new weak emission band at 583 nm. The deprotonated form of this fluorophore exhibits dual fluorescence emission (with maxima at 418 and 583 nm), which we attribute to an excited-state deprotonation process coupled with an ICT process [27].

### 2.3. Fluorescence Intensity Modulation by Addition of NaOH and Followed by UV Light Irradiation

**Figure 5:**
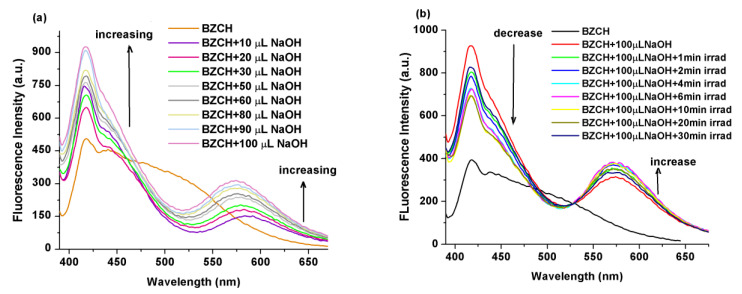
Changes in the emission spectra of BZCH in DMSO solutions upon the gradual addition of NaOH (0.1 N) (**a**) followed by irradiation with 365 nm UV light for various time intervals (**b**). The corresponding absorption maximum was used as the excitation wavelength.

By combining the effects of deprotonation (through the addition of dilute NaOH solutions) and light exposure of the BZCH solution, a stronger enhancement in emission was observed. Specifically, a DMSO solution of BZCH was first titrated with successive small volumes of NaOH (10–100 µL, Figure 5a), followed by exposure to 365 nm UV light irradiation at different time intervals (Figure 5b), and emission spectra were recorded. It can be observed that, during the deprotonation process (Figure 5a), the emission band at 417 nm increases in intensity, and the shoulder at 510 nm, becomes a well-structured emission band with a maximum at 573 nm (for 100 µL NaOH added), whose emission intensity increases with increasing NaOH concentration. Then, the resulting solution (BZCH in DMSO + 100 µL NaOH) was exposed to UV light at 365 nm for different time intervals and, surprisingly, the intensity of the main emission band with maxima at 417 nm decreased, while the main emission band at longer wavelengths (573 nm) increased in intensity (Figure 5b) with increasing irradiation time.

### 2.4. Effects of UV Light Irradiation on the Optical Spectra

**Figure 6:**
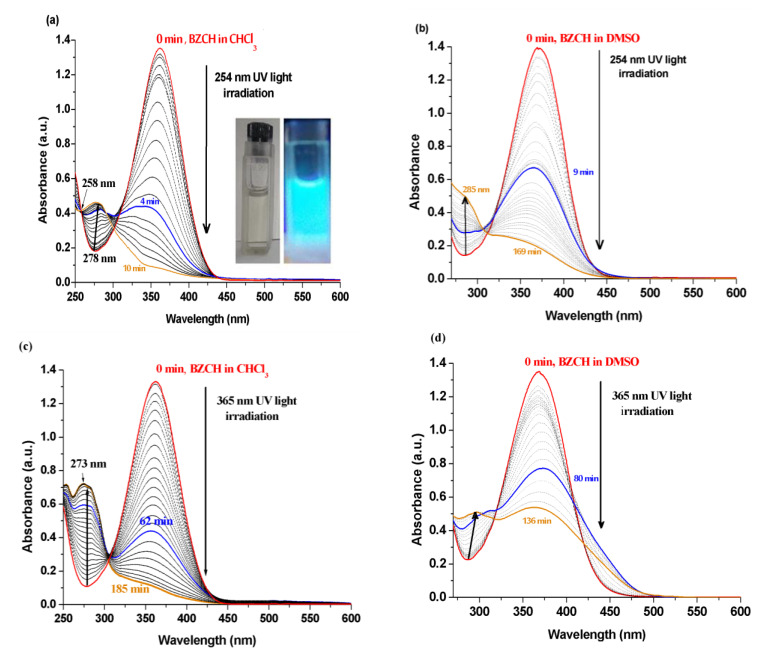
UV-Vis absorption spectral variations of BZCH during irradiation with 254 nm (**a**,**b**) and 365 nm (**c**,**d**) UV light at various intervals of time, in CHCl3 (**a**,**c**) and DMSO (**b**,**d**) solutions. Inset: Photograph of BZCH under daylight and UV light.

Figure 6 shows the UV-Vis absorption spectra of BZCH after being irradiated with 254 and 365 nm UV light, in CHCl_3_ and DMSO solutions for various intervals of time. Before irradiation, BZCH showed absorption maxima at 363 and 369 nm in CHCl_3_ and DMSO solutions, respectively, which were attributed to π-π* transitions of C=C in the chains (Figure 6). After being irradiated with 254 nm UV light, the intensity of the absorption maxima decreases due to the disappearance of the double bond in the chalcone unit, and the intensity of the absorption around 278 nm slightly enhanced with increasing irradiation time from 0 to 185 min (Figure 6a,b). This increase in absorption intensity around 278 nm might be due to the formation of a new structure [28] through a cycloaddition reaction under UV irradiation. It is known that irradiation of molecular structures containing the reactive ketoethylene group (-CO-CH=CH-) with UV light can induce photoisomerization (cis-trans isomerization) and photocyclization between the polymer chains [23] and resulting new structures. Upon UV light irradiation (10 min at 254 nm and 185 min at 365 nm) to the sample in CHCl_3_ solvent, the absorption band from 363 nm almost disappeared (Figure 6a,c). Slow changes in spectral behavior were seen following irradiation with 254 nm UV light in DMSO (Figure 6b) as compared to those observed in chloroform solution (Figure 6a) because DMSO provides a viscous medium that exhibits a certain structural effect, such as resistance to twisting [29].

The photoisomerization of BZCH occurred faster under UV 254 nm irradiation than under UV 365 nm irradiation, as observed for other cyclohexanone derivatives [30]. Moreover, the CHCl_3_ medium (Figure 6a,d) gives more flexibility for the chalcone units to increase the degree of photocrosslinking (PD%) compared to that obtained in the DMSO solution. The PD% values were calculated using the following relation: PD% = (A_0_ − A_T_)/A_0_ × 100, where A_0_ and A_T_ are the absorption intensities due to >C=C< after irradiation times t = 0 and t = T, respectively. Thus, the magnitude of PD% is in the following order: 93.96% (10 min irradiated with 254 nm light in CHCl_3_) > 91.96% (185 min irradiated with 254 nm light in DMSO) > 85.95% (169 min irradiated with 254 nm light in CHCl_3_) > 60.26% (136 min irradiated with 254 nm light in DMSO) for the BZCH sample. These results showed good values compared to other studies for some photoactive cyclohexanone derivatives [31] when the maximum PD% values were 67% and 55% after illumination of samples in THF solution under irradiation with a UV lamp (450 nm) for 30 min.

**Figure 7:**
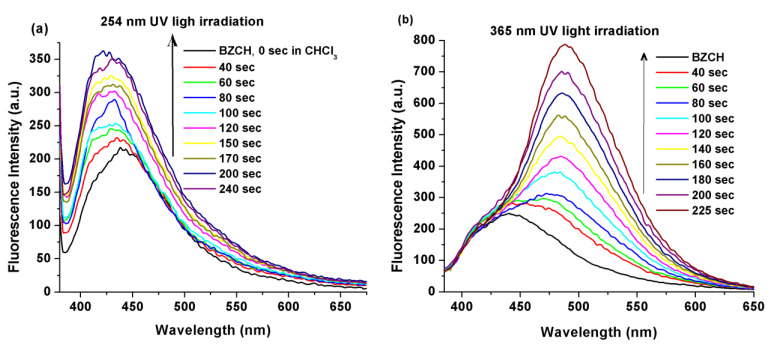

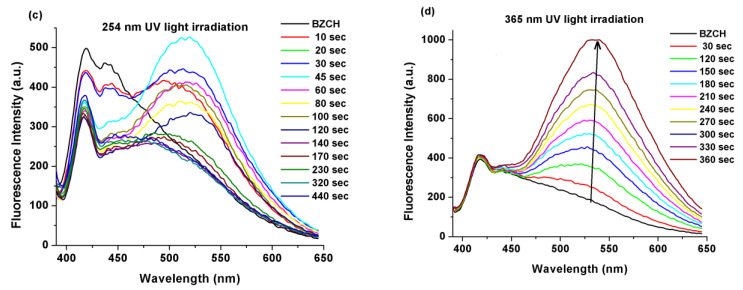
Changes in the fluorescence spectra of BZCH in CHCl_3_ (**a**,**b**) and DMSO (**c**,**d**) solutions, upon irradiation with 254 (**a**,**c**) and 365 nm (**b**,**d**) UV light. The excitation wavelength was set at 362 nm in CHCl_3_ and 370 nm in DMSO solvents.

The BZCH sample exhibited emission bands with maxima at 439 and 417 nm (with a shoulder at 511 nm) in pure CHCl_3_ and DMSO solutions, respectively. In these emission spectra, a 22 nm hypsochromic shift was observed upon going from chloroform (a low polarity medium) to DMSO (a polar medium). As seen in Figure 7a–d, the emission band is much more defined/resolved in chloroform solutions and much broader in DMSO, with a shoulder at 511 nm. Upon analyzing the absorption and fluorescence spectra of BZCH, it was discovered that the Stokes shifts differed in the CHCl_3_ and DMSO solvents, measuring at 50 and 77 nm, respectively. This suggests the existence of weak interactions between the solute and solvent molecules and could also be attributed to a mild tendency for self-quenching and internal charge transfer (ICT) within the system [23,32,33,34,35].

After irradiation with 254 and 365 nm UV light, the fluorescence spectral patterns changed drastically (Figure 7). Thus, upon irradiation with 254 nm UV light, the emission band intensity (λ_max_ = 439 nm, Figure 7a) in CHCl_3_ increased with increasing irradiation time from 0 to 240 sec, but without a shift of the maximum emission. In contrast, in the DMSO solution, after irradiation with 254 nm, a slight increase in the emission band intensity (from 417 nm) was observed, but also an intensification in the intensity for the shoulder at 511 nm, where an unclear increase or decrease in emission intensity occurs during irradiation (Figure 7c). Meanwhile, after irradiation with 365 nm UV light (Figure 7b), a redshift (from 439 nm to 487 nm in CHCl_3_ and from 417 nm to 533 nm in DMSO solution) of the emission band and a significant increase in emission intensity with increasing irradiation time were observed.

## 3. Materials and Methods

2,6-Bis(4-hydroxybenzylidene) cyclohexanone (BZCH) was synthesized as previously reported in the literature [23], and its chemical structure is shown in Figure 1, which was confirmed by the following FTIR and ^1^H NMR data [23]:

FTIR (KBr, cm^−1^): 3358 (-OH), 1640 (C=O), 1580 (C=C); ^1^H NMR (DMSO-D6, δ ppm); 1.7 (quintet, 2H, J = 5.5 Hz γ to the keto group of cyclohexanone), 2.9 (triplet, 4H, J = 5.2 Hz, β to the keto group of cyclohexanone), 6.8–7.4 (doublet, 8H aromatic, J = 8.6 Hz), 7.6 (s, 2H, -CH=), 9.9 (s, 2H, -OH). All spectroscopic-grade solvents were purchased and used without further purification.

Infrared spectra were studied using an FTIR spectrophotometer (Bruker Optik GmbH, Ettlingen, Germany), and ^1^H NMR data were recorded with a Bruker Avance Neo instrument (Bruker BioSpin, Rheinstetten, Germany).

The absorption and fluorescence spectra for the BZCH sample were measured in different solvents using an Analytic Jena 210+ spectrophotometer (Analytik Jena, Germany) and an LS55 photoluminescence spectrofluorometer (Perkin Elmer, Inc., Waltham, MA, USA) equipped with a xenon lamp as the light source and quartz cuvettes with a 1 cm optical path length. All measurements were performed at room temperature.

For the photoirradiation experiments, a UV hand lamp (NH-15, Herolab, Germany) with two UV wavelengths of 254 and 365 nm, was utilized. The photoirradiation was performed with different exposure times using a quartz cuvette with a 1 cm optical path length. The distance between the sample and the lamp was kept constant at 1 cm throughout the measurements. The acid/base controlled response (acidochromic behavior) experiments were performed by titrating the sample in DMSO solution with dilute HCl/NaOH solutions (0.1 N) and measuring the absorption and emission spectra after each solution was stabilized for 2 min.

The absorption, excitation, and emission spectra of BZCH in DMSO and CHCl_3_ solutions are displayed in Figure S2 in the Supplementary Materials. Fluorescence spectra of the BZCH sample were acquired using an excitation wavelength that corresponded to the wavelength of the absorption maximum (363 nm in CHCl_3_ and 369 nm in DMSO) with a slit width of 5 nm and for different excitation wavelengths (Figure S3). Additionally, we performed stability studies in solution. After 8 h of spectral monitoring in the absence of light, the absorption bands in DMSO (Figure S4a) and CHCl_3_ (Figure S4b) did not change significantly, with only a slight increase in absorbance resulting from solvent evaporation.

Multiparametric regression analysis was performed by using the Origin 6.0 software program to achieve the correlation coefficients. The values for the solvent descriptors, including the KAT (*α*, *β*, π*) [24], Catalán (*SA, SB, SdP, SP*) [25], and Laurence (*DI, ES, α*_1_*,* β_1_) [26] solvent parameters were taken from literature sources and are listed in Table 1 and Table 2.

## 4. Conclusions

Photo-/basichromism and polarity sensitivity of the BZCH probe were characterized using absorption and fluorescence spectra in fifteen solvents with different characteristics. Three multiple linear regression solvatochromic models were used to quantify solvent–solute intermolecular interactions. The results showed that the polarizability/dipolarity parameter of the solvent plays a more significant role than its acidity or basicity in the solvatochromic behavior of the sample. This was confirmed by using the KAT, Catalán, and Laurence empirical solvatochromic scales. The Catalán scale exhibited a higher correlation compared to the Laurence and KAT methods. The addition of NaOH to BZCH in DMSO solution resulted in a gradual disappearance of the absorption intensity at around 369 nm, accompanied by an increase in a new absorption band with a maximum at 514 nm. Fluorescence-enhancement effects were observed after exposure to 254 and 365 nm UV light in both CHCl_3_ and DMSO solvents.

## Figures and Tables

**Table 1:** The UV-Vis absorption maxima (λ_max_ and ${\tilde{\nu }}_{\exp}^{\max}$ ), electronic transition energies (E_T_ (kJ mol^−1^)) of BZCH and KAT {*α*, *β*, and π*} empirical solvent parameters [24].

No.	Solvents	λ_max_ (nm)	${\tilde{\nu }}_{exp}^{max}\;(\mathrm{cm}{-}^{1})$	E_T_ ^a^ (exp)	*α*	*β*	π*
1.	Dioxane	356	28,089.88	336.02	0.00	0.37	0.55
2.	Toluene	351	28,490.02	340.81	0.00	0.11	0.54
3.	EtAc	356	28,089.88	336.02	0.00	0.45	0.54
4.	THF	358	27,932.96	334.14	0.00	0.55	0.58
5.	DCE	354	28,248.58	337.92	0.00	0.00	0.807
6.	1-Butanol	246.5; 376	26,595.74	318.15	0.79	0.88	0.47
7.	2-Propanol	374	26,737.96	319.85	0.76	0.95	0.48
8.	1-Propanol	246; 374	26,737.96	319.85	0.78	0.85	0.52
9.	Methanol	245; 369.5	27,063.59	323.74	0.93	0.62	0.60
10.	Acetone	356.5	28,050.49	335.55	0.08	0.48	0.71
11.	DMF	367.0	27,247.95	325.95	0.00	0.69	0.87
12.	ACN	241; 354	28,248.58	337.92	0.19	0.31	0.75
13.	DMSO	369	27,173.91	325.06	0.00	0.76	1.00
14.	NMP	369	27,100.27	324.18	0.00	0.00	0.92
15.	DMAc	368.5	28,089.88	324.18	0.00	0.76	0.88

^a^ transition energies (E_T_) were calculated from the conversion of the corresponding wavelengths of the absorption maxima (λ_max_) of BZCH using the following relation,$\;E_{T}/{\mathrm{kJmol}}^{-1}=\mathrm{hcN}/\lambda_{\max}$. Notations: λ_max_—wavelength of absorption maximum; ${\tilde{\nu }}_{\exp}^{\max}$—wavenumber of absorption maximum; EtAc—ethyl acetate; THF—tetrahydrofuran; DCE—1,2-dichlorethane; DMF–N,N—dimethylformamide; ACN—acetonitrile; DMSO—dimethylsulfoxide; NMP—N-methyl pyrrolidone; DMAc—dimethylacetamide.

**Table 2:** Empirical Catalán {*SA, SB, SP*, and *SdP*} and Laurence {*DI, ES, α*_1_, and *β*_1_} solvent parameters [25,26].

No.	Solvent	*SA*	*SB*	*SP*	*SdP*	*DI*	*ES*	*α*_1_	*β*_1_
1.	Dioxane	0	0.444	0.737	0.312	0.77	0.36	0.00	0.44
2.	Toluene	0	0.128	0.782	0.284	0.86	0.20	0.00	0.15
3.	EtAc	0	0.542	0.656	0.603	0.71	0.51	0.00	0.52
4.	THF	0	0.591	0.714	0.634	0.75	0.47	0.00	0.58
5.	DCE	0.030	0.126	0.771	0.742	0.80	0.74	0.00	0.00
6.	1-Butanol	0.341	0.809	0.674	0.655	0.74	0.75	0.65	0.67
7.	2-Propanol	0.283	0.830	0.633	0.808	0.72	0.77	0.68	0.65
8.	1-Propanol	0.367	0.782	0.658	0.748	0.71	0.77	0.53	0.68
9.	Methanol	0.605	0.545	0.608	0.904	0.64	0.84	1.00	0.54
10.	Acetone	0.000	0.475	0.651	0.907	0.69	0.78	0.04	0.49
11.	DMF	0.031	0.613	0.759	0.977	0.78	0.87	0.00	0.69
12.	ACN	0.044	0.286	0.645	0.974	0.67	0.84	0.23	0.37
13.	DMSO	0.072	0.647	0.830	1.000	0.84	1.00	0.00	0.71
14.	NMP	0.024	0.613	0.812	0.959	0.83	0.80	0.00	0.76
15.	DMAc	0.028	0.650	0.763	0.987	0.79	0.85	0.00	0.75

**Table 3:** Resulting coefficients (×10^3^ cm^−1^) from the multiparametric regression analysis of ${\;\nu \;\text{˜}}_{exp}^{\max}$ and E_T_ of the BZCH as a function of the KAT, Catalán, and Laurence (Equations (1)–(3)) solvent scales.

KAT	Y_0_	a_1_ (*α*)	b_1_ (*β*)	c_1_ (π*)		^a^ R^2^
${\tilde{\nu }}_{\exp}^{\max}$	29.50	−1.19	−0.943	−1.786		0.775
E_T_	352.96	−14.08	−11.39	−21.21		0.767
**Catalán**	**Y_0_**	**a_2_ (*SA*)**	**b_2_ (*SB*)**	**c_2_ (*SP*)**	**d_2_ (*SdP*)**	**R^2^**
${\tilde{\nu }}_{\exp}^{\max}$	31.32	−1.858	−1.976	−3.062	−0.434	0.961
E_T_	375.44	−22.01	−23.91	−37.55	−4.97	0.959
**Laurence**	**Y_0_**	**a_3_ (*DI*)**	**b_3_ (*ES*)**	**c_3_ (*α*_1_)**	**d_3_ (*β*_1_)**	**R^2^**
${\tilde{\nu }}_{\exp}^{\max}$	31.96	−3.948	−0.490	−1.503	−1.393	0.843
E_T_	387.20	−50.83	−7.78	−16.00	−20.24	0.944

^a^ Squared correlation coefficient (R^2^).

**Table 4:** Percentage contributions of the KAT, Catalán, and Laurence solvent parameters to the observed solvatochromism of the BZCH sample.

Parameter Sets	% *α*	% *β*	% π*	
KAT	30.36	24.06	45.57	
Catalán	**% *SA***	**% *SB***	**% *SP***	**% *SdP***
	25.34	26.95	41.77	5.92
Laurence	**% *α*_1_**	**% *β*_1_**	**% *DI***	**% *ES***
	7.75	22.03	62.45	7.75

## Data Availability

Not applicable.

## Supplementary Materials

The supporting information can be downloaded at: https://www.mdpi.com/article/10.3390/ijms24065286/s1.
