# Supplementary material for: Solvatochromism, Acidochromism and Photochromism of the 2,6-Bis(4-hydroxybenzylidene) Cyclohexanone Derivative
Source: Int J Mol Sci. 2023 Mar 9;24(6):5286. doi: 10.3390/ijms24065286 (PMC10049195; doi:10.3390/ijms24065286)
Supplement: Supplementary file 1 [file ijms-24-05286-s001.zip › ijms-2209831-supplementary.pdf]

Supplementary Materials
for
Solvatochromism, acidochromism and photochromism of the 2,6-bis(4-hydroxybenzylidene) cyclohexanone derivative

Mihaela Homocianu[1], Diana Serbezeanu, Vlad Bubulac Tachita
"Petru Poni" Institute of Macromolecular Chemistry, 41A, Grigore Ghica Voda Alley, 700487, Iasi, Romania

1) Calculated absorption maxima for the BZCH sample *versus* corresponding experimental data

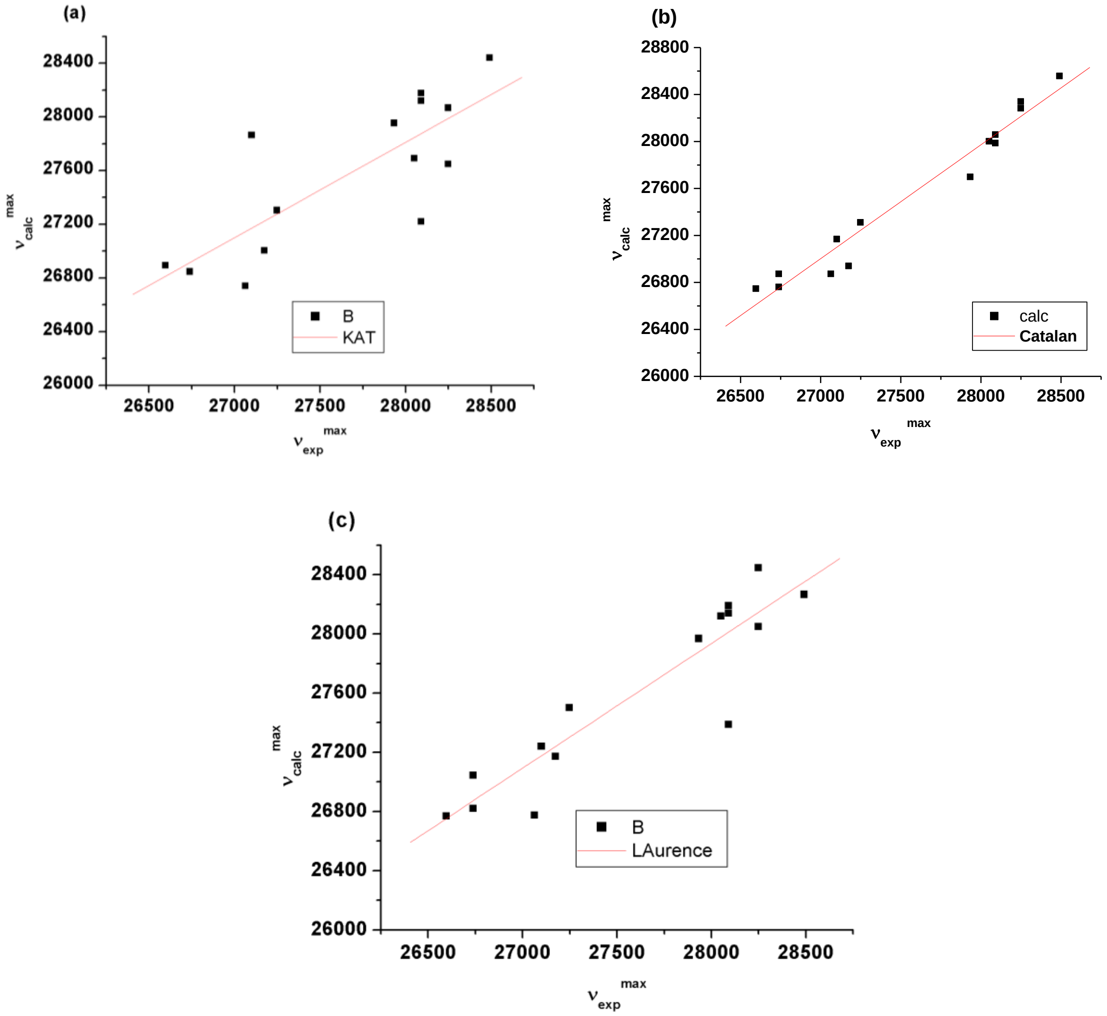

Figure S1. Calculated absorption band of **BZCH** sample obtained by (a) Kamlet-Taft, (b) Catalan and (c) Laurence regression analysis, plotted against the corresponding experimentally observed absorption band positions.

---
[1] Corresponding author: Tel.: 0232 217 454.

2) Analysis of the results from multiple regression fitting of absorption spectral data using different parameter sets

Analysis of the results from multiple regression fitting of absorption spectral data using different parameter sets.

(a) Kamlet- Abraham-Taft (KAT) parameter set:

$$\tilde{\nu}_{\mathrm{max}} = (27.79 \pm 0.02) - (1.17 \pm 0.03)\alpha \qquad (R^2=0.441) \qquad (S1)$$

$$\tilde{\nu}_{\mathrm{max}} = (27.77 \pm 0.03) - (0.76 \pm 0.02)\alpha - (0.13 \pm 0.05)\beta \qquad (R^2=0.221) \qquad (S2)$$

$$\tilde{\nu}_{\mathrm{max}} = (29.26 \pm 0.03) - (1.70 \pm 0.02)\alpha - (1.96 \pm 0.05)\pi \qquad (R^2=0.640) \qquad (S3)$$

$$\tilde{\nu}_{\mathrm{max}} = (29.50 \pm 0.04) - (1.19 \pm 0.03)\alpha - (0.94 \pm 0.03)\beta - (1.78 \pm 0.06)\pi \qquad (R^2=0.775) \qquad (S4)$$

The fit to the only α solvatochromic parameter has a low R^2 value (0.441) (Eq. S1). However, the addition of the π polarity parameter results in a substantial increase in R^2 (0.640) (Eq. S2). When all three KAT solvent parameters {α, β, π} are included in the fit, R^2 moderately increases (0.764, Eq. S3), indicating that the β term is not significant in fitting the data.

(b) Catalán parameter set:

$$\tilde{\nu}_{\mathrm{max}} = (27.80 \pm 0.01) - (2.32 \pm 0.07)S\mathrm{A} \qquad (R^2=0.441) \qquad (S5)$$

$$\tilde{\nu}_{\mathrm{max}} = (28.92 \pm 0.02) - (2.61 \pm 0.03)S\mathrm{B} \qquad (R^2=0.771) \qquad (S6)$$

$$\tilde{\nu}_{\mathrm{max}} = (26.62 \pm 1.8) - (1.25 \pm 2.5)S\mathrm{P} \qquad (R^2=0.018) \qquad (S7)$$

$$\tilde{\nu}_{\mathrm{max}} = (28.49 \pm 0.5) - (1.26 \pm 0.6)Sd\mathrm{P} \qquad (R^2=0.206) \qquad (S8)$$

$$\tilde{\nu}_{\mathrm{max}} = (28.82 \pm 0.01) - (1.16 \pm 0.04)S\mathrm{A} - (2.15 \pm 0.03)S\mathrm{B} \qquad (R^2=0.859) \qquad (S9)$$

$$\tilde{\nu}_{\mathrm{max}} = (31.17 \pm 0.06) - (1.88 \pm 0.03)S\mathrm{A} - (2.13 \pm 0.02)S\mathrm{B} - (3.19 \pm 0.08)S\mathrm{P} \qquad (R^2=0.940) \qquad (S10)$$

$$\tilde{\nu}_{\mathrm{max}} = (31.32 \pm 0.05) - (1.85 \pm 0.02)S\mathrm{A} - (1.97 \pm 0.02)S\mathrm{B} - (3.06 \pm 0.06)S\mathrm{P} - (0.43 \pm 0.01)Sd\mathrm{P}$$

(R^2=0.961 (S11)

The addition of either the SB (Eq. S9) or SP parameter (Eq. S10) alone leads to an increase in R^2 values compared to R^2 resulting from Eqs. S5-8 where only one solvent parameter was used. However, the presence of the SdP parameter provides the largest increase in R^2 (Eq. S11). When

all four parameters are included, R^2 is the highest of all, showing that these terms are significant in describing the dependence of the transition energy on solvent parameters.

(c) Laurence parameter set:

$$\tilde{\nu}_{max} = (28.18 \pm 0.20) - (0.62 \pm 0.20)DI - (1.51 \pm 0.07)ES \qquad (R^2=0.273) \quad (S12)$$

$$\tilde{\nu}_{max} = (28.70 \pm 0.02) - (1.07 \pm 0.02)\alpha_1 - (1.65 \pm 0.04)\beta_1 \qquad (R^2=0.740) \quad (S13)$$

$$\tilde{\nu}_{max} = (31.62 \pm 0.01) - (3.77 \pm 0.02)DI - (1.54 \pm 0.02)\alpha_1 - (1.61 \pm 0.03)\beta_1 \qquad (R^2=0.824) \quad (S14)$$

$$\tilde{\nu}_{max} = (31.96 \pm 0.01) - (3.94 \pm 0.01)DI - (0.49 \pm 0.02)ES - (1.50 \pm 0.03)\alpha_1 - (1.39 \pm 0.04)\beta_1 \qquad (R^2=0.843) \quad (S15)$$

Eliminating the ES contributions in Eq. S14 leads in a negligible decrease in the correlation coefficient, from R^2=0.843 to 0.824 indicating a slight contribution of the ES parameter.

3) Absorption, excitation, and emission spectra of BZCH in DMSO and $CHCl_3$ solvents

(a) (b)

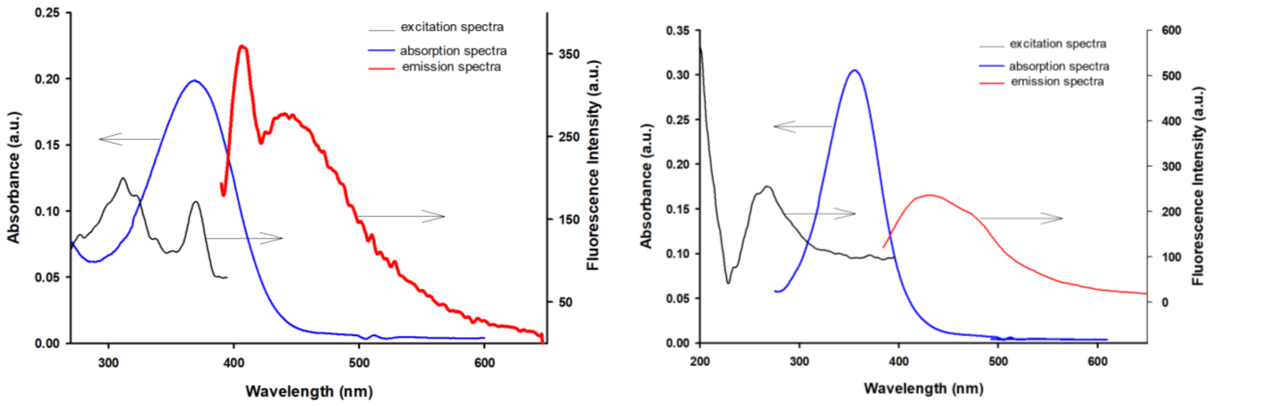

Figure S2. Absorption, excitation, and emission spectra of BZCH in (a) DMSO and (b) $CHCl_3$ solvents.

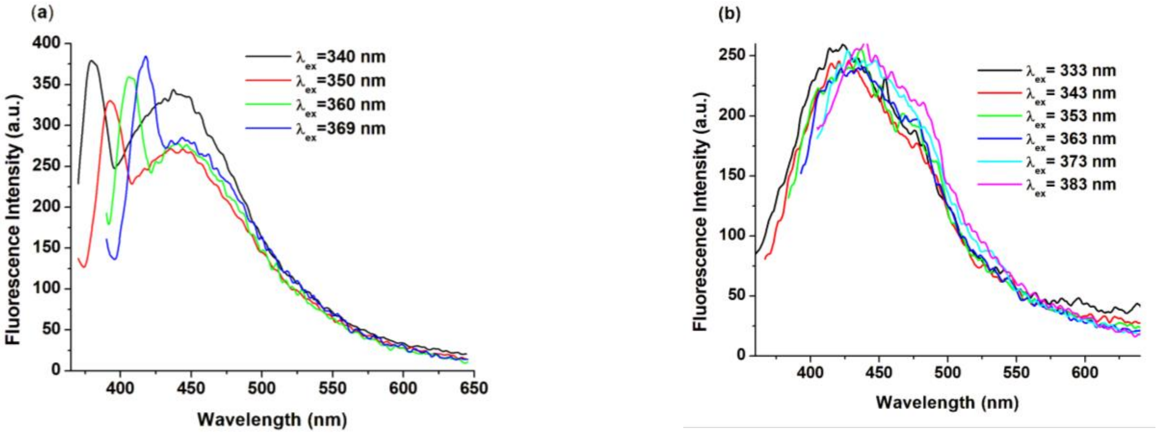

Figure S3. Fluorescence spectra of a dilute solution of BZCH in (a) DMSO and (b) $CHCl_3$ at different excitation wavelengths.

4) Stability studies of BZCH in DMSO and $CHCl_3$ solutions.

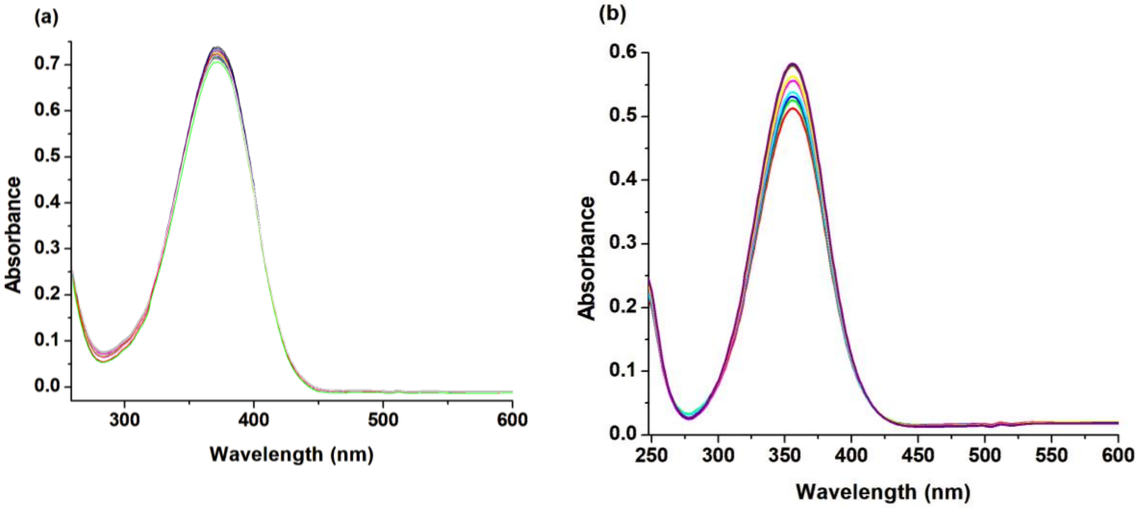

Figure S4. Monitoring of absorption over 8 h in the absence of light for solutions of BZCH in DMSO (a) and $CHCl_3$ (b).
